# Establishment of an Immune Cell Infiltration Score to Help Predict the Prognosis and Chemotherapy Responsiveness of Gastric Cancer Patients

**DOI:** 10.3389/fonc.2021.650673

**Published:** 2021-07-09

**Authors:** Quan Jiang, Jie Sun, Hao Chen, Chen Ding, Zhaoqing Tang, Yuanyuan Ruan, Fenglin Liu, Yihong Sun

**Affiliations:** ^1^ Department of General Surgery, Zhongshan Hospital, Fudan University, Shanghai, China; ^2^ Human Phenome Institute, Fudan University, Shanghai, China; ^3^ Department of Biochemistry and Molecular Biology, School of Basic Medical Sciences, Fudan University, Shanghai, China

**Keywords:** gastric cancer, immune cell infiltration, prognosis factors, chemotherapy, immunotherapy

## Abstract

The immune microenvironment plays a critical role in tumor biology. The molecular profiles of immune components and related genes are of tremendous value for the study of primary resistance to immune checkpoint blockers (ICBs) for gastric cancer (GC) and serve as prognostic biomarkers to predict GC survival. Recent studies have revealed that tumor immune cell infiltration (ICI) is an indicator of the survival and responsiveness to chemotherapy in GC patients. Here, we describe the immune cell landscape based on the ESTIMATE and CIBERSORT algorithms to help separate GC into 3 ICI clusters using the unsupervised clustering method. Further in-depth analyses, such as differential expression gene (DEG) analysis and principal component analysis (PCA), help to establish an ICI scoring system. A low ICI score is characterized by an increased tumor mutation burden (TMB). The combination of the ICI score and TMB score better predicts the survival of GC patients. Analyses based on public and our own database revealed that the ICI scoring system could also help predict the survival and chemotherapy responsiveness of GC patients. The present study demonstrated that the ICI score may be an effective prognostic biomarker and predictive indicator for chemotherapy and immunotherapy.

## Introduction

Gastric cancer (GC) is a heterogeneous disease that is found worldwide, and it is ranked as the fifth most common cancer and the third leading cause of cancer-related death worldwide (1). Despite all of the effort devoted to improving the curative effect in the last decade, the treatment outcomes of GC remain unsatisfactory. As concluded in previous studies, only 30% of GC is diagnosed at an early stage, and the 5-year survival for pTNM stage groups divided based on the American Joint Committee on Cancer (AJCC) is between 68 and 80% for stage I, 46 and 60% for stage II,8 and 30% for stage III and 5% for stage IV (2). Compared with the data from the National Cancer Database (NCDB) Research Unit of the American College of Surgeons, GC patents in eastern Asia are diagnosed at a relatively early stage and enjoy better survival. However, there is much room for improvement in treatment outcomes (3). The heterogeneity of GC is reflected in the complexity of its classification. Based on the different phenotypes under the microscope, GC is traditionally divided into 3 major histological subtypes according to Lauren’s classification, namely, intestinal, diffuse and mixed types (4). Although regarded as a relatively old and crude typing method, Lauren’s classification system remains widely used because of its contingency and continence. The most widely acknowledged pathological system is the World Health Organization (WHO) classification, which divides GC into four main types based on the predominant histological patterns: tubular adenocarcinoma, papillary adenocarcinoma, mucinous adenocarcinoma and poorly cohesive carcinoma (5). The last 10 years witnessed the establishment of different molecular classifications of GC. The Cancer Genome Atlas (TCGA) project, which divides GC into four subtypes, EBV-positive (EBV), microsatellite-unstable (MSI), genomically stable (GS) and chromosomal instability (CIN), has attracted the most attention (6).

The immune microenvironment plays a critical role in tumor biology. The molecular profiles of the immune components and related genes provide tremendous value for the study of primary resistance to immune checkpoint blockers (ICBs) for GC and serve as prognostic biomarkers to predict GC survival. Several recent studies proposed immune-related gene signatures for risk stratification and the predicting of clinical outcomes in different cancers (7–9). Tumors are composed of stromal and immune components. Multiple recent studies shed light on the vital role that tumor-infiltrating immune cells play in development, progression, recurrence and drug resistance (10, 11). For example, T cells are the first step in the elimination of cancer and recognize and kill tumor cells and secrete anti-cancer factors that inhibit the function of immune-regulating cells, such as T-reg cells (12). Tumor-associated macrophages (TAMs) and tumor-infiltrating neutrophils (TINs) help immune escape *via* the self-expression of PD-L1 to inhibit the anti-tumor function of T-cells, which ultimately leads to an unfavorable prognosis of GC (13, 14). Our previous studies also revealed correlations of specific immune cells and their corresponding clinical value in GC, including prognosis prediction, chemotherapy resistance and tumor dissemination (15–17).

The present study initially described the immune cell infiltration (ICI) landscape using CIBERSORT and ESTIMATE algorithms to quantify the tumor components and proportion of tumor-infiltrating cells. Based on the ICI results, we separated GC into three subtypes according to the ICI patterns. Differentially expressed genes (DEGs) derived from the comparison between the 3 groups were acquired to help further classify GC into two gene groups. The gene clusters were further used to establish a digitized ICI score system under the consideration of somatic mutation variations. The system exhibited the potential to predict the response to immunotherapy and chemotherapy for GC patients.

## Materials and Methods

### Data Source

The transcriptome profiles, clinical data and simple nucleotide variations (SNVs) of GC patients were derived from The Cancer Genome Atlas (TCGA) Genomic Data Commons Data Portal (https://portal.gdc.cancer.gov/, December 10, 2020). The transcriptome profiles and clinical data of ACRG, GSE84437, GSE26253 and GSE26899 were also acquired from the Gene Expression Omnibus database (GEO) (https://www.ncbi.nlm.nih.gov/geo/, December 10, 2020). The tracking IDs of all the GC patients are listed in **Supplementary Table 1**. To render the comparability of the TCGA cohort with the other GEO cohorts, the expression profile (FPKM values) of TCGA-STAD datasets was transformed into TPMs (transcripts per million kilobases) as previously reported (18). RNA-seq and survival data of 29 GC patients enrolled in clinical trial concerning neoadjuvant chemotherapy were also acquired. Mandard tumor regression grade (TRG) was applied as the criteria for calculating the chemotherapy response of patients (19). This system classifies pathologic response as follows: TRG 1 (complete regression/fibrosis with no evidence of tumor cells), TRG 2 (fibrosis with scattered tumor cells), TRG 3 (fibrosis and tumor cells with a dominance of fibrosis), TRG 4 (fibrosis and tumor cells with a dominance of tumor cells), and TRG 5 (tumor without evidence of regression). Grade 1-2 was defined as major response while Grade 3-5 as minor response.

### Examination of the Immune Spectrum for Consensus Clustering

Based on the merged transcriptional data derived from the 3 datasets, CIBERSORT and ESTIMATED algorithms were used to evaluate the 22 immune cell proportions and immune and stromal scores for each sample. The effective data were further acquired using the filtering process of the CIBERSORT algorithm. Data from 853 cases were effective and selected for further investigations. Hierarchical agglomerative clustering was performed based on the ICI pattern of each sample. The “ConsensuClusterPlus” package from R software was used to perform the unsupervised clustering “Pam” method based on Euclidean and Ward’s linkage analysis (20).

### Acquisition of DEGs Based on the ICI Clustering System

Transcriptional data were compared between the 3 different ICI clusters by setting significance cut-off criteria to p < 0.05 (adjusted) and absolute fold-change > 1. All of the genes acquired were defined as DEGs.

### Calculations of ICI Scores

This process was initiated by performing unsupervised clustering to calculate each DEG value of TCGA GC patients. DEG values that positively correlated with the cluster signature were defined as ICI gene signature A, and negatively correlated DEG values were defined as ICI gene signature B. The Boruta algorithm was used for dimension reduction of ICI gene signatures A and B, and principal component 1 was extracted as the signature score using PCA. A method similar to the gene expression grade index was used to define the ICI score of each patient, and the following formula:

ICI score=∑PC1A−∑PC1B

### Evaluation of the Sensitivity of Chemotherapeutic Agents and Immunotherapy Efficacy

To examine the sensitivity of each sample to different chemotherapeutic agents, the “pRRophetic” package in R software (3.6.1) was used to predict the half-maximal inhibitory concentration (IC50) of chemotherapy drugs for the comparison of the high- and low-risk groups in training cohort. This algorithm was previously published and widely used in multiple studies (21). The online tool Tumor Immune Dysfunction and Exclusion (TIDE) (http://tide.dfci.harvard.edu) was used to predict the immunotherapeutic responses of each sample based on the transcriptome profiles. Since only data with log2(RPKM+1) values were applicable for the TIDE calculations, the TCGA and Zhongshan cohorts were finally chosen for further analysis. The TIDE score was compared between the high and low ICI score groups. A lower TIDE score indicated a relatively better response to immunotherapy.

### RNA Extraction and Sequencing

Formalin fixation and paraffin embedding tissues of 29 patients were acquired from the pathology department of Zhongshan Hospital after obtaining the patient’s informed consents. Total RNA was extracted from recently cut 10 μm FFPE sections using the miRNeasy FFPE kit (Qiagen, Valencia, CA) according to the manufacturer’s protocol, using 1–4 sections (10–40 μm) per case depending on assay. RNA yield and quality were determined by UV absorption on a NanoDrop 1000 spectrophotometer and fragment size was analyzed using the RNA 6000 Nano assay (Agilent Technologies, Santa Clara, CA) run on the 2100 Bioanalyzer. DV200 values representing the percentage of RNA fragments above 200 nucleotides in length were determined according to Agilent and Illumina recommended protocols. To determine the minimal amount of tissue needed to yield adequate RNA quantity for library preparation, RNA yield per 10 μm section number was tested. Based on the test results, one or two 10 μm sections of breast FFPE specimens were used for RNA isolation. RNA quality was assessed using DV200 values and cases with DV200 more than 27% were included for library preparation. After Library preparation, Sequencing was performed on a Hi-Seq 2500 using a 100 cycle, single read protocol with a depth of approximately 90 million reads per sample. Following initial sequencing, 3 of the 6 libraries were repooled and independently sequenced. Base call files were converted to fastq format using Bcl2Fastq (Illumina). All RNA-seq reads were aligned to the human reference genome (GRCh38, release 84) using STAR (version 2.5.2b).

## Results

### The Spectrum of Infiltrative Immune Cells in the Tumor Microenvironment of GCs.

**Figure 1:**
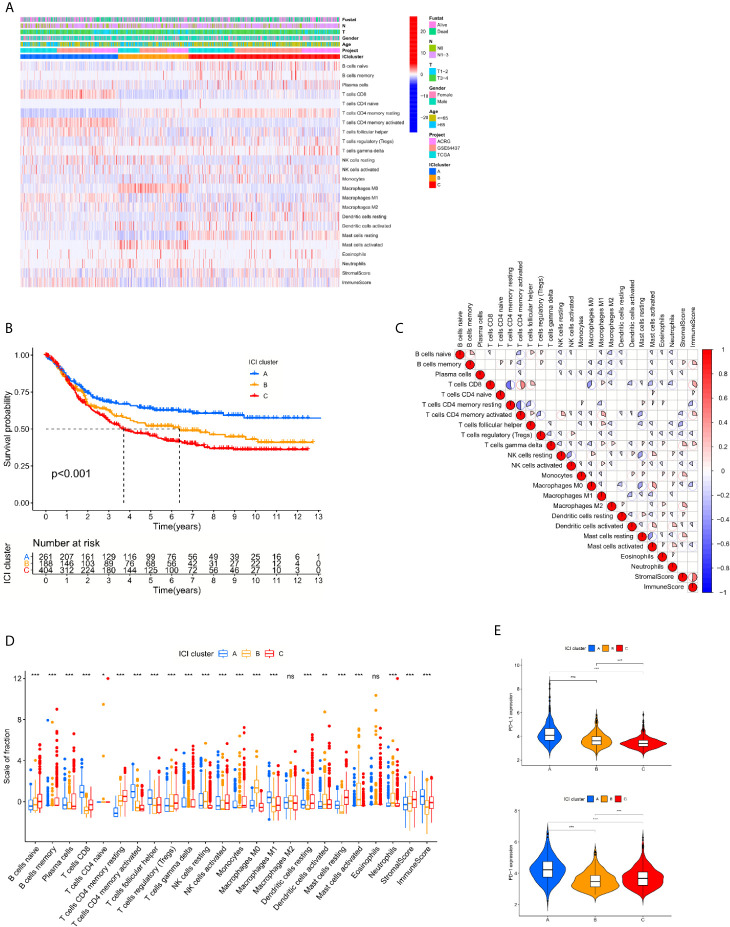
The spectrum of infiltrative immune cells in the tumor microenvironment of GC. **(A)** Unsupervised clustering of 22 immune cells derived from CIBERSORT algorithm in 3 GC datasets. The patients were divided into 3 cohorts based on the spectrum of infiltrative immune cells. **(B)** Log rank test shown by Kaplan-Meier curves indicated a significant overall survival difference between the different immune cell-infiltrating (ICI) classes, p <0.001. **(C)** The correlations between 22 immune cells derived from CIBERSORT algorithm. **(D)** The fraction variations of tumor-infiltrating immune cells in three ICI clusters. Immune score and stromal score of three ICI clusters based on the ESTIMATE algorithm were also compared. (Kruskal-Wallis test. *p < 0.05; **p < 0.01; ***p < 0.001; ns, not significantly different). **(E)** Expression variations of PD-L1 and PD1 among different ICI cohorts (Kruskal-Wallis test, *p < 0.05; **p < 0.01; ***p < 0.001; ns, not significantly different).

Studies were initially performed using the CIBERSORT and ESTIMATE algorithms to quantify the levels of immune and stromal components in each sample of training cohort (**Supplementary Tables 2** and **3**). A total of 853 cases were selected for further process based on the CIBERSORT filtering process of 1108 patients. The patients were divided into 3 cohorts based on the unsupervised clustering of 22 immune cells derived from the CIBERSORT algorithm in 3 GC datasets (**sFigure 1**). ICI cluster A presented the best survival, and ICI cluster C exhibited the worst survival (**Figures 1A–C**). To further examine the hidden biological function variations between the 3 cohorts, the proportions of each immune cell type were also compared. The results indicated that most of the 22 immune cells varied, except M2 macrophages and eosinophils. We reviewed the details of infiltrating immune cells in each ICI cluster and found that anti-cancer immune cells, such as CD8 T cells, activated memory CD4 T cells and M1 macrophages, were elevated in ICI cluster A, and the volume of immune-regulating cell-like regulatory T cells (Tregs) was increased in ICI cluster C. The component variations partially explained the survival variations between different ICI clusters. The ESTIMATE algorithm results indicated that the immune components were more enriched in ICI cluster A and the stromal components were more enriched in ICI cluster C (**Figure 1D**). These results might also explain the prognosis discrepancy between different clusters. To further examine the potential predictive value of the ICI clusters, the expression of PD-L1/PD-1 was compared between different groups. The Kruskal-Wallis test revealed that the expression of PD-L1 and PD-1 was ranked highest in the ICI cluster A (**Figure 1E**).

### Immune Gene Subtypes Establishment

**Figure 2:**
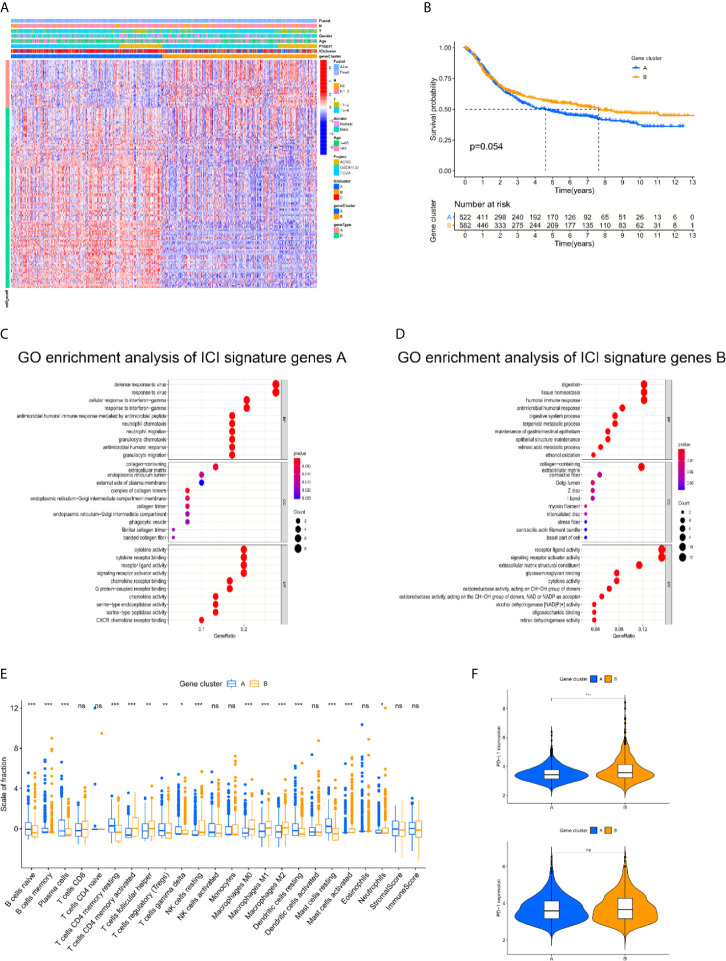
The landscapes of immune related genes and subtype establishment. **(A)** Unsupervised clustering of differentially expressed genes derived from three ICI clusters helped divide patients into two groups, namely gene clusters A and gene clusters B. **(B)** The log rank test shown by Kaplan-Meier curves indicated an approaching statistical significance for overall survival comparison between two gene clusters. (p = 0.054). **(C, D)** Comparison of Gene Ontology (GO) enrichment analysis between the two ICI-relevant signature genes. **(E)** The fraction variations of tumor-infiltrating immune cells in two gene clusters. Immune score and stromal score of two gene clusters based on the ESTIMATE algorithm were also compared. (Kruskal-Wallis test. *p < 0.05; **p < 0.01; ***p < 0.001; ns, not significantly different). **(F)** Expression variations of PD-L1 and PD1 among different gene cohorts (Kruskal-Wallis test, *p < 0.05; **p < 0.01; ***p < 0.001; ns, not significantly different).

To examine the hidden biological characteristics of each ICI cluster, differential analyses were performed to identify the DEGs between these subtypes. Each of the 3 clusters was compared to each other to obtain 3 groups of DEGs. All of the genes were gathered to acquire 152 DEGs (**Supplementary Table 4**, **sFigure 2**). Unsupervised clustering and the Boruta algorithm of these 152 DEGs derived from the three ICI clusters helped divide the patients into two groups, namely, gene cluster A and gene cluster B (**Figure 2A**, **sFigure 3**). The log rank test showed by Kaplan-Meier curves indicated an approaching statistical significance for overall survival comparison between the two gene clusters. (p = 0.054) (**Figure 2B**). Comparison of Gene Ontology (GO) enrichment analysis between the two ICI-relevant signature genes revealed that the two clusters were enriched in different pathways (**Figures 2C, D**). Some of the pathways enriched in gene cluster A, such as NEUTROPHIL CHEMOTAXIS and NEUTROPHIL MIGRATION, are a sign of worse prognosis (14). The fraction variations of tumor-infiltrating immune cells in the two gene clusters were significant and the immune score and stromal score of the two gene clusters based on the ESTIMATE algorithm were also revealed to be statistically variated (Kruskal-Wallis test. *p < 0.05; **p < 0.01; ***p < 0.001; ****p < 0.0001; ns: not significantly different). To further examine the hidden biological function variations between the 2 cohorts, the proportions of each immune cell type were compared. We reviewed the details of infiltrating immune cells in the two gene clusters and found that anti-cancer immune cells, such as CD8 T cells, activated memory CD4 T cells and M1 macrophages, were elevated in gene cluster B, and the volume of immune-regulating cell-like regulatory T cells (Tregs) was increased in gene cluster A. The component variations partially explained the survival variations between different gene clusters. However, the ESTIMATE algorithm results revealed that immune and stromal scores were not different between groups. These results seem inconsistent with the CIBERSORT algorithm (**Figure 2E**). We speculated that the quality, but not the quantity, of immune components play a more important role in gene clusters. To further examine the potential predictive value of the gene clusters, the expression of PD-L1/PD-1 was compared between different groups. The Kruskal-Wallis test revealed that the expression of PD-L1 was significantly higher in gene cluster B, while PD-1 showed no difference (**Figure 2F**).

### Construction and Validation of the ICI Score Classification System

**Figure 3:**
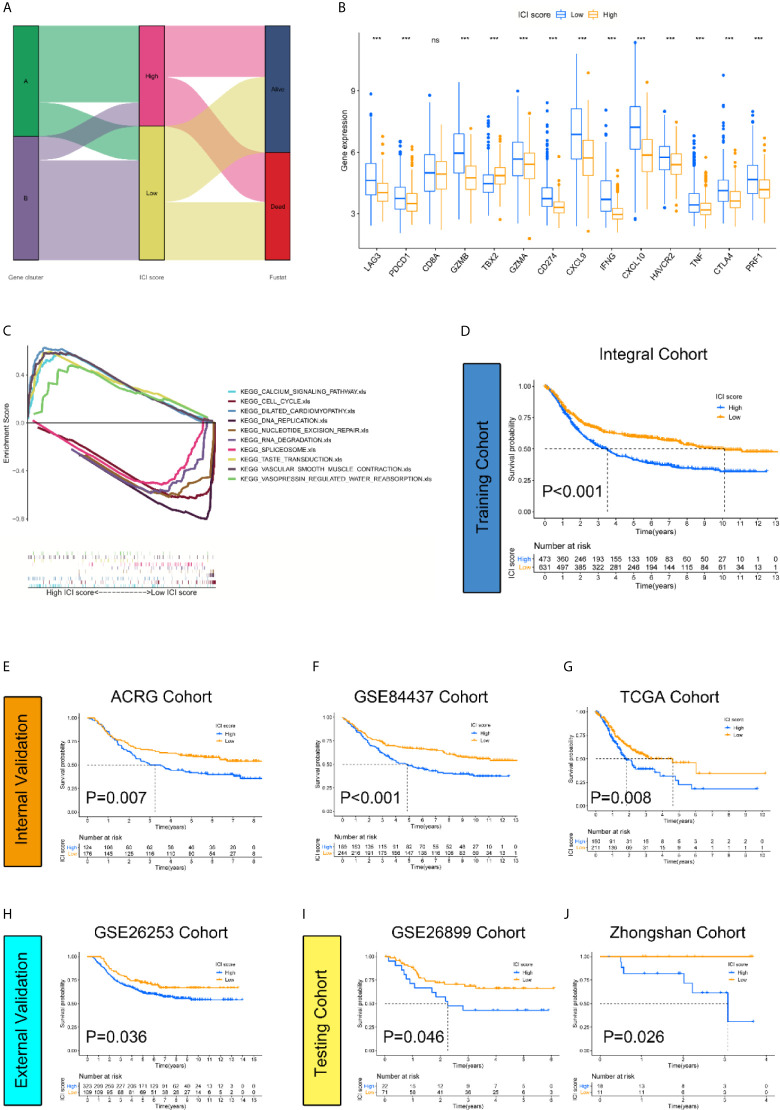
Construction and validation of the ICI score classification system. **(A)** Alluvial diagram revealed the correlations between different ICI clusters, ICI scores, and survival outcomes. **(B)** Expression level of immune-checkpoint-relevant genes were significantly higher in low ICI score group. (Kruskal-Wallis test. ***p < 0.001; ns, not significantly different). **(C)** GSEA KEGG enrichment analysis. CALCIUM_SIGNALING_PATHWAY, CELL_CYCLE, DILATED_CARDIOMYOPATHYECM, DNA_REPLICATION and NUCLEOTIDE_EXCISION_REPAIR pathways are enriched in high ICI score cohort while RNA_DEGRADATION, SPLICEOSOME, TASTE_TRANSDUCTION, VASCULAR_SMOOTH_MUSCLE_CONTRACTION and VASOPRESSIN_REGULATED_WATER_REABSORPTION pathways are enriched in low ICI score cohort. **(D–J)** The log rank test shown by Kaplan-Meier curves indicated a better overall survival for low ICI score groups in all datasets, including training cohort {(Integral Cohort, p = 0.007) **(D)**, internal validation cohort (ACRG Cohort, p < 0.001) **(E)**, (GSE84437 Cohort, p < 0.001) **(F)**, (TCGA Cohort, p =0.008) **(G)**, external validation cohort (GSE26253 Cohort, p = 0.036) **(H)** and testing cohort (GSE26899 Cohort, p = 0.046) **(I)**, (Zhongshan Cohort, p =0.026) **(J)**}.

To quantify the infiltrative immune cell landscape, principal component analysis (PCA) was performed to acquire two aggregate scores. One score was ICI score A (ISA) from the ICI signature gene, and the other score was ICI score B (ISB) from ICI signature gene B. The ISA and ISB of each sample were calculated as the sum of relevant scores, which was defined as the ICI score. The correlations of the gene clusters, ICI score clusters and patient survival status are presented in **Figure 3A**. We compared the expression levels of immune checkpoint-relevant genes (CD274, HAVCR2, PDCD1, CTLA4, and LAG3) and immune activation-relevant genes (CD8A, CXCL10, CXCL9, GZMA, GZMB, PRF1, IFNG, TBX2, and TNF) between the high and low ICI score subgroups to investigate their predictive value for immune checkpoint therapy. The results revealed that most of the targets were elevated in the low ICI score cohort, except CD8A and TBX2 (**Figure 3B**). GSEA Kyoto Encyclopedia of Genes and Genomes (KEGG) enrichment analysis revealed that the CALCIUM_SIGNALING_PATHWAY, CELL_ CYCLE, DILATED_CARDIOMYOPATHYECM, DNA_ REPLICATION and NUCLEOTIDE_EXCISION_REPAIR pathways were enriched in the high ICI score cohort, and the RNA_DEGRADATION, SPLICEOSOME, TASTE_TRANSDUCTION, VASCULAR_SMOOTH_ MUSCLE_CONTRACTION and VASOPRESSIN_ REGULATED_WATER_REABSORPTION pathways were enriched in the low ICI score cohort (**Figure 3C**). To examine the prognostic value of the ICI score, the patients were stratified into two groups based on ICI scores using the optimal cut-off value acquired by the Youden index calculation algorithm. The Integral Cohort consisting 1108 cases (4 patients were excluded for survival loss) were set as training cohort and the survival analysis revealed that the lower ICI score group presented with better prognosis (**Figure 3D**). The ICI score also exhibited good performance in prognosis-predicting in internal validation cohorts including ACRG, GSE84437 and TCGA (**Figures 3E–G**). We further examine the efficacy of ICI score with GSE26253 Cohort composed of 432 patients. The results also indicated a better prognosis of the lower score group (**Figure 3H**). To finally prove the practicality of ICI score, GSE26899(n=93) and Zhongshan Cohorts(n=29) were applied as testing cohorts. The results were highly consistent, proving the superiority of the ICI score for prognosis prediction (**Figures 3I, J**).

**Figure 4:**
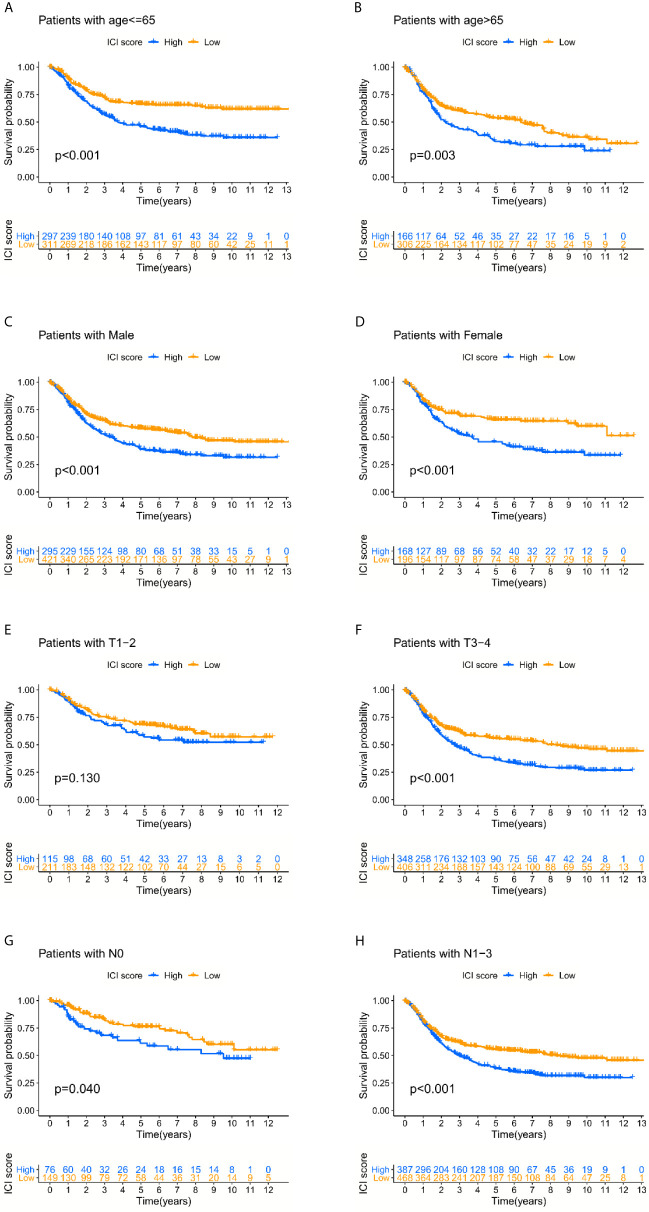
The prognosis-predicting efficacy of the ICI score classification system in different clinical subgroups. **(A–D)** Lower ICI score could help predict the better overall survival regardless of the age and gender. **(E–H)** The predictive efficacy was weakened in early-stage cases compared with the advanced stage cases.

The prognostic value of the ICI score was further tested based on the different clinical data. The results indicated that a lower ICI score helped predict better overall survival regardless of age and sex, and the predictive efficacy was weakened in early-stage cases compared to advanced-stage cases. It might highlight the defects of the ICI score as an overall survival predictor (**Figures 4A–F**).

### The Interrelations Between the ICI Score and Somatic Mutation Variants (SNVs)

**Figure 5:**
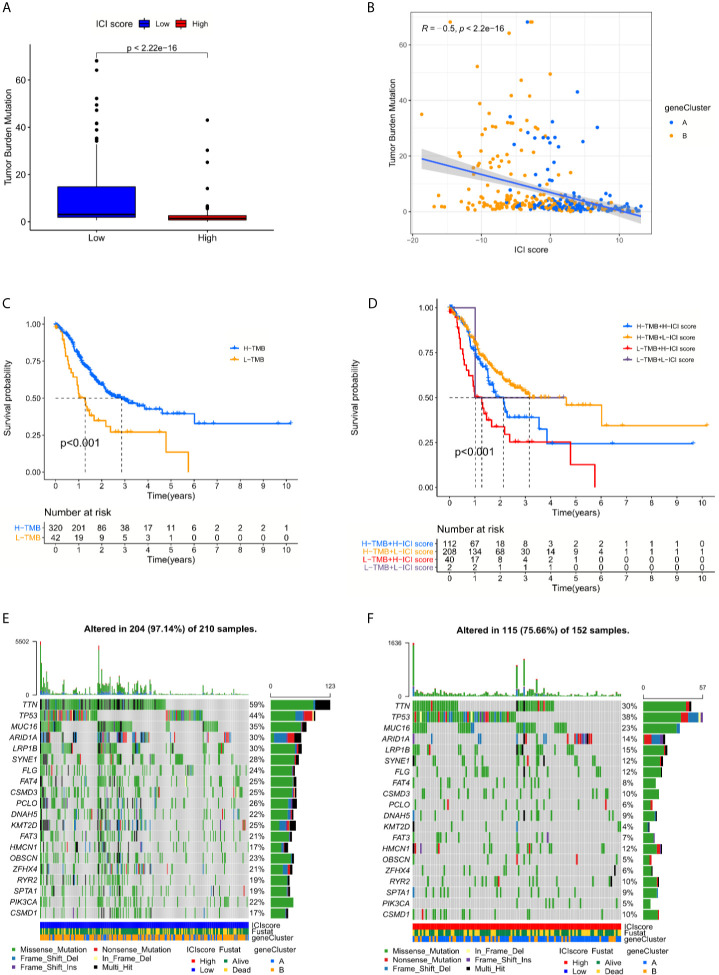
The interrelations between the ICI score and somatic mutation variants (SNVs) **(A)** Tumor mutation burden (TMB) variations between the high and low ICI score groups. (Wilcoxon test, p < 0.0001) **(B)** The positive correlation between ICI scores and mutation load in TCGA-STAD cohort was shown by scatterplots. (Spearman text, p < 0.0001). **(C)** The log rank test shown by Kaplan-Meier curves indicated a better overall survival for high TMB score groups. (p < 0.001). **(D)** The combination of TMB and ICI score helped predict the overall survival. **(E, F)** The SNVs spectrum was constructed based on different ICI scores clusters, blue for low and red for high.

Numerous studies supported the hypothesis that accumulating somatic mutation variants trigger the immune system to produce anti-cancer cells. Increased tumor mutation burden was also identified as an indicator of an improved response to PD-1 blockade and prolonged progression-free survival (22). Taking the somatic mutation variants into account, we investigated the intrinsic correlation between the TMB and ICI scores to elucidate the genetic imprints of each ICI subgroup. Based on the SNV data derived from TCGA-STAD, the TMB values for each sample with ICI scores were calculated. Then TMB values of the high and low ICI score clusters were compared using the Wilcoxon test. The results indicated that patients in the low ICI score subgroup showed a significantly higher TMB than patients in the low ICI score subgroup (**Figure 5A**). Further Spearman correlation analysis also confirmed that the ICI score negatively correlated with the TMB values (**Figure 5B**). The log rank test shown by Kaplan-Meier curves indicated better overall survival for the high TMB score groups (**Figure 5C**). The combination of TMB and ICI score helped predict overall survival (**Figure 5D**). All of these results suggest that the ICI score may be an important predictive factor to indicate the response to immunotherapy. To evaluate the driver mutations of each ICI score cohort, the mutated genes were listed using maftools. The result was not significant regardless of the type or frequency of the mutated genes, which suggests the present of more unknown mechanisms (**Figures 5E, F**).

### Drug Sensitivities Prediction Using the ICI Score Classification System

**Figure 6:**
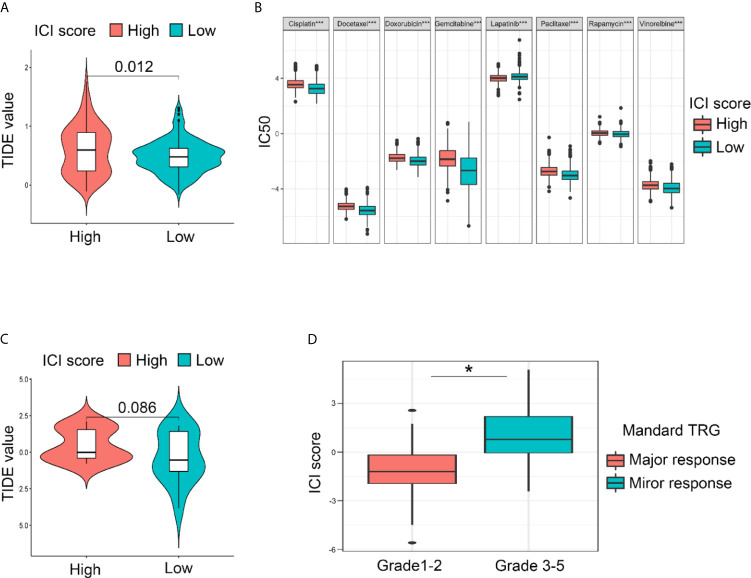
Drug sensitivities prediction using the ICI score classification system based on different cohorts. **(A)** The lower ICI score group presented with lower TIDE score compared with the higher ICI score group, indicating that the lower ICI score group is more sensitive to the immune check point therapy in TCGA Cohort. **(B)** The IC50 for GC-concerning chemotherapeutics were compared between low and high ICI score groups. Most of the IC50 was lower in low ICI score group except for lapatinib in Integral Cohort. (Kruskal-Wallis test. ***p < 0.001; ns, not significantly different). **(C)** As for the Zhongshan Cohort, the TIDE value was not significantly different despite a tendency of decreasing in low ICI score group. **(D)** A lower ICI score was also observed in patients diagnose as major response after neoadjuvant chemotherapy, confirming the value of ICI score for chemotherapy response prediction in Zhongshan Cohort. (Kruskal-Wallis test. *p < 0.05).

To examine the clinical application value of the ICI score, the TIDE value and drug IC50 for each sample were calculated. The online tool TIDE was first used to investigate the effectiveness of immune checkpoint (PD-1 and CTLA-4) inhibitors in immunotherapy in the high and low ICI score groups of TCGA Cohort. The results revealed that the low ICI score group harbored a lower TIDE score, which indicates the potential value of the ICI score classification system to help identify patients who will benefit from immune checkpoint therapy (**Figure 6A**). As for Zhongshan Cohort, the TIDE value was not significantly different despite a tendency of decreasing in low ICI score group (**Figure 6C**). Based on the pRRophetic algorithm and the drugs “pRRophetic” package, we analyzed 8 GC chemotherapy drugs that are used in GC treatment to predict the IC50 of each sample (cisplatin, doxorubicin, doxorubicin, gemcitabine, lapatinib, paclitaxel, rapamycin and vinorelbine) in two ICI score groups. The results revealed that the IC50 values for most drugs were lower in the low ICI score group, except lapatinib. These results confirm that the ICI score classification system is a good predictor for the successful application of chemotherapy (**Figure 6B**). Since all the patients of the Zhongshan Cohort had accepted neoadjuvant chemotherapy, we compared the ICI score between the major response and mirror response patients. The results also indicated a lower ICI score for the major response patients (**Figure 6D**). It suggested that ICI score was worth further exploration for its value as chemotherapy response predictor. The flowchart of the whole study was presented in **Figure 4**.

## Discussion

The past 30 years have witnessed rapid progress in the treatment of GC, and the survival rate of GC patients has increased rapidly, especially in countries such as Japan and Korea (23). The reason was largely due to the improvement in early diagnosis, which was attributed to the wide promotion of gastrointestinal endoscopy tests. The application of multimodal treatments, including neoadjuvant chemotherapy, surgery, postoperative radiotherapy and chemotherapy, also contributed to the treatment progress. However, the overall survival rate of GC remains unsatisfactory (1). ICB treatment is a rising star in cancer therapy. The efficacy of PD-L1/PD-1 treatment was observed in multiple cancer types, especially lung cancer and melanoma (24, 25). However, most of the clinical trials targeting the PD-L1/PD-1 axis produced negative results (26). However, it is unfair to directly reject the use of ICB treatment in GC because PD-L1/PD-1 is one of the many ICB pathways. A solid prediction system for the guidance of PD-L1/PD-1 blocker application is urgently required. The results of PD-L1/PD-1 immunohistochemistry (IHC) are one of the most important selection criteria for patients who may benefit from ICB treatment (26, 27). However, the positive rate of PD-L1 expression in GC was only 14.3–29.6%, and its drug response predictive efficacy was limited (28–30). Microsatellite instability (MSI) is recognized as a distinct subgroup of GC by The Cancer Genome Atlas (TCGA) and the Asian Cancer Research Group (ACRG) classification (31). MSI-H GCs are significantly related to high tumor infiltrating lymphocytes and high expression of PD-L1 in a positive manner, regardless of the IHC and transcription levels. On the basis of these hypotheses, recent studies suggested the use of MSI-H status as a biomarker for treatment selection with immune checkpoint inhibitors (32–34). However, the detection methods of MSI status remain controversial, which limits the application of MSI status for drug sensitivity prediction. An immune checkpoint score system for prognostic evaluation and adjuvant chemotherapy selection in gastric cancer was established recently and provides a new selection for drug sensitivity prediction (35).

The present study analyzed the ICI spectra in a meta-cohort of 1108 GC samples and divided the sample into three distinct immune subtypes. The results indicated that most of the 22 immune cells were variable, except M2 macrophages and eosinophils. We reviewed the details of infiltrating immune cells in each ICI cluster and found that anti-cancer immune cells, such as CD8 T cells, activated memory CD4 T cells and M1 macrophages, were elevated in ICI cluster A, and the volume of immune-regulating cell-like regulatory T cells (Tregs) was increased in ICI cluster C. These results partially explain the survival variations between the different ICI clusters and highly correlate with previous studies (8, 9, 26). However, the pre-existing results of ICB-related studies reminded us of the defect of merely focusing on a single gene or immune component. We assumed that the integrated spectra of the ICI profiles and immune-related gene expression patterns would be an effective method for the prediction of immunotherapy and chemotherapy. To address this problem, we further performed differential analyses to determine the differentially expressed genes (DEGs) between different ICI clusters. Unsupervised clustering and Boruta algorithm of these DEGs derived from the three ICI clusters helped divide patients into two groups, namely, gene cluster A and gene cluster B. We reviewed the details of infiltrating immune cells in two gene clusters and found that anti-cancer immune cells, such as CD8 and CD4 activated memory T cells and M1 macrophages, were elevated in gene cluster B and that the volume of immune-regulating cell-like T cells regulatory (Tregs) was increased in gene cluster A. However, the ESTIMATE algorithm results revealed that immune and stromal scores were not different between groups. These results seem inconsistent with the CIBERSORT algorithm. We hypothesize that the quality, but not the quantity, of immune components matters in gene clusters.

Based on the previous results, principal component analysis (PCA) was performed to quantify the infiltrative immune cell landscape, namely, the ICI score. The value of the system for prognosis prediction was further confirmed by validation and texting cohorts. In particular, the Zhongshan Cohort, which was composed of patients receiving neoadjuvant chemotherapy, also presented with consistent results despite a relative short follow-up interval. An integrated ICI score at the genome level also revealed significant variant frequency differences in immune checkpoint-relevant genes (CD274, HAVCR2, PDCD1, CTLA4, and LAG3) and immune activation-relevant genes (CD8A, CXCL10, CXCL9, GZMA, GZMB, PRF1, IFNG, TBX2, and TNF) between the high and low ICI score subgroups. The high predictive efficacy of ICB markers supported the potential value of the ICI score system for clinical applications. TMB was also used alone or in combination with the ICI score to predict the survival of the TCGA-STAD cohort. The result was interesting because the high TMB group exhibited better survival than the low TMB group. This conclusion was totally different from other cancer types, such as breast cancer and neck squamous cell carcinoma (36, 37). This result was found in previous GC-related studies. Somatic mutations of MUC16 and TNN correlated with better survival in gastric cancer (38). The reason why high TMB helps prolong the survival of GC may be cautiously explained by the triggering of the immune system by the accumulation of SNVs. However, the hypothesis is incompatible considering the negative correlation between the ICI score and TMB. More intensive studies are urgently needed to elucidate the mechanism.

To examine the clinical application value of the ICI score, the TIDE value and drug IC50 were calculated for the training cohort and Zhongshan cohort. The online tool TIDE was first used to investigate the effectiveness of immune checkpoint (PD-1 and CTLA-4) inhibitors in immunotherapy in high and low ICI score groups. Since only data with log2(RPKM+1) values were applicable for the TIDE calculations, the TCGA and Zhongshan cohorts were finally chosen to be proper for further analysis. The results revealed that the low ICI score group harbored a lower TIDE score in TCGA cohort. However, the TIDE value was not significantly different despite a tendency of decrease in low ICI score group in Zhongshan Cohort. The value of the ICI score classification system for immunotherapy response prediction remained uncertain. Based on the pRRophetic algorithm and the drugs “pRRophetic” package included, we analyzed 8 GC chemotherapy drugs that are used in GC treatment to predict the IC50 of each sample (cisplatin, doxorubicin, doxorubicin, gemcitabine, lapatinib, paclitaxel, rapamycin and vinorelbine) for the Integral cohort. The results revealed that the IC50 values for most drugs were lower in the low ICI score group, except laptinib. The patients enrolled in Zhongshan cohort had all received neoadjuvant chemotherapy and the results of each patient’s responsiveness to chemotherapy can be measured by pathological examination after surgery. Mandard TRG was the most acknowledged criteria (39). We made the comparison between the major and mirror response groups and the results also confirmed that lower ICI score presented with better treatment response to chemotherapy. These results confirmed that the ICI score classification system might be a good instructor for proper application of chemotherapy.

In facts, previous studies have also tried to develop a gene sets or score system to help find out the patients whom might benefit from chemotherapy. Wu et al. established 2 prognostic models designated as stable gene risk scores of OS (SGRS-OS) and stable gene risk scores of PFI (SGRS-PFI) consisting of 18 and 21 genes respectively for GC. The gene set-based system was claimed to harbor the capability of chemotherapy response predictions (40). However, the biggest defects for the study were the lack of validation cohorts. Yan et al. also established an immune cell signature based on ssGSEA analysis and regression model and also claimed its efficacy of chemotherapy response predictions in GC (41). The signature consisted only two GEO cohorts(n=732) and set PFS as the endpoints. The efficacy of this model for chemotherapy response prediction were actually not directly proved. Similar study has also been reported in other types of tumors. Sui et al. developed an immune cell infiltration-based immune score model to predict prognosis and chemotherapy effects in breast cancer. The study enrolled 5112 patients for analysis and established the signature by using proportions of immune cells (CIBERSORT) to perform Lasso and Cox regression analysis directly. Compared with the study, the methods we used to establish the score system were totally different. Our study indeed did not try to quantify a specific immune cell, but wished to stratify samples into different modules based on the immune spectrum variations elucidated by CIBERSORT algorithm. The ICI score was established based on PCA analysis of the DEGs between the clusters. The reason we designed this analyzing process was to reduce the infidelity of CIIBERSORT results revealed by previous research (42). But it was hard to tell which methods were better since both of our studies achieved fairly good results. Compared with breast cancers, an efficient predictor was more urgently needed for GC due to its relative low response rate to chemotherapy. Our study may make a little bit contribution to that study fields.

Limitations inevitably exist in this study and the most significant one is the fidelity of CIBERSORT results. Transcriptome-based cell-type quantification methods for immuno-oncology are indeed all facing similar questionings (43–45). The computer-based algorithms can introduce a technical bias in a basis matrix towards the microarray platform used for transcriptome profiling, resulting in lower deconvolution accuracy for samples that are profiled using different platforms. Furthermore, basis matrices to date have been created using expression data solely from healthy subjects, which can further introduce biological bias that could affect deconvolution accuracy and limit their applicability to samples from patients with disease (46–48). Vallania et al. conducted CIBERSORT analysis of two basis matrices, IRIS and LM22, both created using only one microarray platform and healthy samples The result showed significant heterogeneity in deconvolution results between different microarray platforms, and lower discriminatory power for distinguishing blood and solid tissue samples when obtained from a patient instead of a healthy control. It reminded us the existing defects of the computational-based algorithm. As a workaround, the study also revealed that leveraging heterogeneity across multiple datasets increased cell-mixture deconvolution accuracy and reduced biological and technical biases (42). Inspired by this research, we integrated 3 GC-related datasets as training cohort to conducted CIBERSORT analysis. The 3 datasets were the most acknowledged ones according to previous studies and all were composed of more than 300 patients. We believed the process could increase the fidelity of results. The following analysis of validation and testing cohorts did prove the high application value of the established ICI score system. Another limitation of the study was that the ICI score could only be obtained from the transcriptome profiling of surgical specimen in GC. To fix the problem, we may find solutions from the field of liquid biopsy. The liquid biopsy concept was introduced for circulating tumor cells (CTC) 10 years ago and rapidly extended to circulating tumor DNA (ctDNA) and other tumor derived products such as circulating cell-free RNA (noncoding and messenger RNA), extracellular vesicles, or tumor-educated platelets (49–52). The non-invasive concepts rendered liquid biopsy unparalleled charm and advantages. Its application value lies in tumor occurrence and progression monitoring, systemic therapy response prediction and early detection of metastatic disease (53). As a kind of circRNAs detectable in plasma, Hsa_circ_0000745 was involved in development processes of (GC) and may serve as promising diagnostic marker for GC in combination with CEA (54). Hsa_circ_0000190 was revealed to be down-regulated in gastric cancer tissues and plasma samples from patients with gastric cancer and correlated with tumor diameter, lymphatic metastasis, distal metastasis, and TNM stage (55). Circulating tumor DNA (ctDNA) is usually only a small portion of circulating free DNA and has recently emerged to predict recurrence or relapse in several cancer types. The Japanese MONSTAR-SCREEN study performed a serial ctDNA assay involving 540 patients with advanced solid tumors, of whom 133 had gastrointestinal (GI) tumors (48 patients had gastric tumors). Published results showed that ctDNA levels in GI tumors were significantly higher compared to non-GI tumors. Interestingly, one-third of the alterations detected in tumors were detected only in ctDNA, not in the tissue sample (53, 56). In a prospective trial that has enrolled 93 patients with resectable gastric cancer, patients with CTCs ≥ 5/7.5 mL detected in postoperative blood samples had significantly inferior disease-free survival (DFS) and overall survival (OS) than those with a smaller number of CTCs (57). Liquid biopsy has also been proven to be helpful in different solid malignancies for detecting resistance to chemotherapy and targeted therapy. Exosomal Ribosomal Protein S3 (RPS3) secreted by cisplatin-resistant tumor cells could be taken up by cisplatin-sensitive cells and thus become chemo resistant (through activation of the PI3K-Akt-cofilin-1 signaling pathway), indicating its potential value for proper chemotherapeutics selection (58). Based on peripheral blood plasma specimens from 40 GC patients received HER2-targeted therapy, the dynamic surveillance of plasma ctDNA genomic features provided instructive information for determining the effeteness of this targeted drug (59). Back to our research, we have a vision whether the combination analysis of the ICI score and markers in plasma may boost the prediction efficacy and render practicality to our study. After reviewing the literatures, we were delighted to find that efforts have been paid by recent publications. Wang et al. established an eight-circRNA assessment model for predicting biochemical recurrence in prostate cancer and revealed distinct immune landscape variations between the high and low risk score groups based on CIBERSORT results. More importantly, two of eight circRNA’s (circ_14736 and circ_17720) were also proved detectable in exosomes of patients’ plasma and could also serve the similar function, indicating its value of being used as liquid biopsy markers (60). Inspired by that, we may try to find markers, involved or not involved in our study, detectable in plasmas to serve as indicator for prognosis and chemotherapy response. If the patients were prone to relapse based on the ICI score, we will conduct a more active liquid biopsy evaluation to achieve early detection of recurrence. As for the chemotherapy response prediction, the patients who are diagnosed as resistant to chemotherapeutics based on ICI score evaluations could also be subject to active surveillance with liquid biopsy. We believed that the combination applications of ICI score and liquid biopsy could enhance its practicability.

In conclusion, we described the ICI landscape of GC and revealed the spectrum of tumor immune response regulation in GC. In-depth study of the immune component and related genes helped establish an ICI score, which was a good predictor of chemotherapy and immunotherapy. The development of the ICI score may help the clinical selection of proper patients who may benefit from chemotherapy and immunotherapy.

## Data Availability Statement

The original contributions presented in the study are included in the article/**Supplementary Material**. Further inquiries can be directed to the corresponding authors.

## Ethics Statement

The studies involving human participants were reviewed and approved by Ethics Committee of Zhongshan Hospital Affiliated to Fudan University. The patients/participants provided their written informed consent to participate in this study. Written informed consent was obtained from the individual(s) for the publication of any potentially identifiable images or data included in this article.

## Author Contributions

QJ performed most of the data analyses; JS, HC and CD performed some of the analyses; ZT, YR, FL, and YS contributed to the design of the analyses; Q.J. wrote the manuscript. QJ, JS, and HC contributed equally to this work.

## Funding

This work was supported by grants from the National Natural Science Foundation of China (Grant Nos. 81872425, 31670806, 81972228, 81872346, 31770855, 82073245 and 82072679).

## Conflict of Interest

The authors declare that the research was conducted in the absence of any commercial or financial relationships that could be construed as a potential conflict of interest.

## Supplementary Material

Supplementary Figure 1 Consensus matrixes of all GC samples for each k (k = 2–5), displaying the clustering stability using 1000 iterations of hierarchical clustering.

Supplementary Figure 2 Venn diagram illustrating the number of DEGs among the two ICI clusters.

Supplementary Figure 3 Consensus matrixes of GC cohorts for each k (k = 2–5), displaying the clustering stability using 1000 iterations of hierarchical clustering based on the DEGs acquired.

Supplementary Figure 4 Flowchart of this study.
