# Supplementary figures and images for: Establishment of an Immune Cell Infiltration Score to Help Predict the Prognosis and Chemotherapy Responsiveness of Gastric Cancer Patients
Source: Front Oncol. 2021 Jul 9;11:650673. doi: 10.3389/fonc.2021.650673 (PMC8299334; doi:10.3389/fonc.2021.650673)

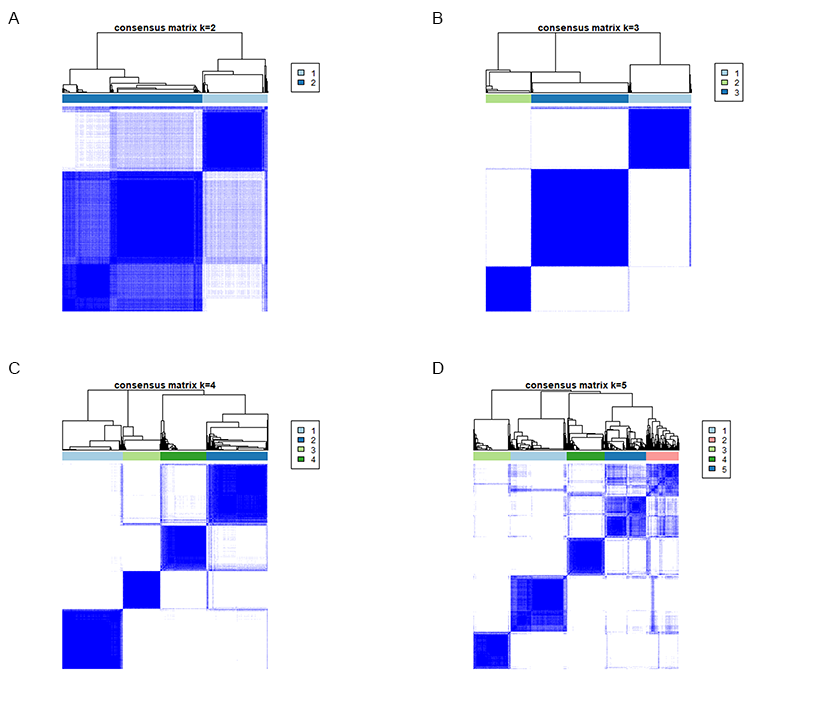

Supplement: Supplementary Figure 1 — Consensus matrixes of all GC samples for each k (k = 2–5), displaying the clustering stability using 1000 iterations of hierarchical clustering. [file Image_1.tif]

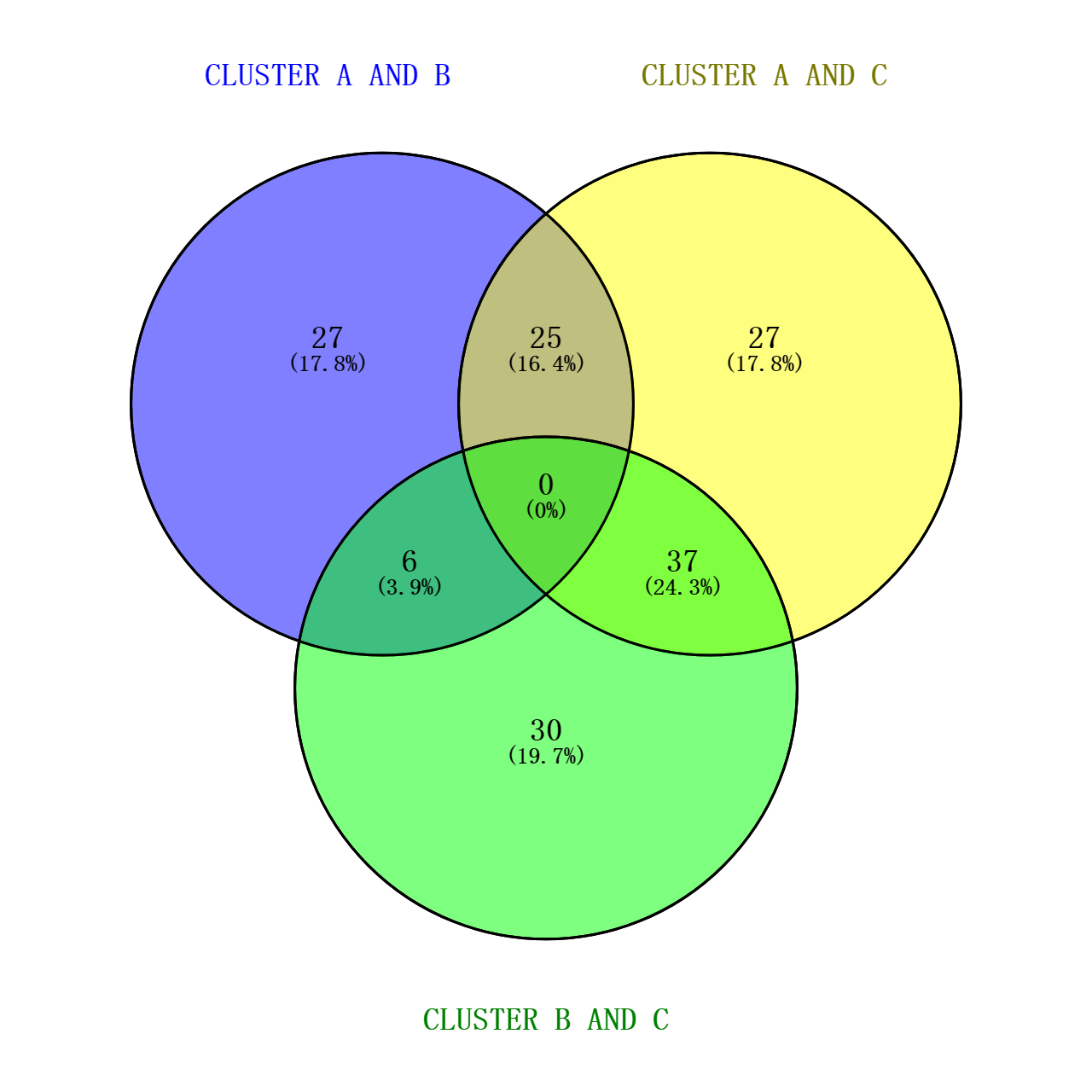

Supplement: Supplementary Figure 2 — Venn diagram illustrating the number of DEGs among the two ICI clusters. [file Image_2.png]

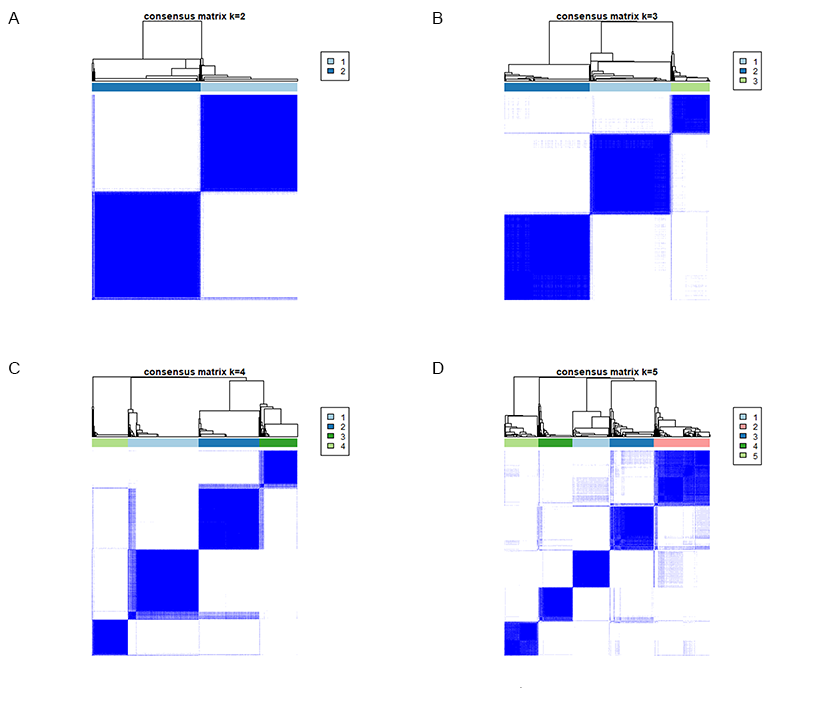

Supplement: Supplementary Figure 3 — Consensus matrixes of GC cohorts for each k (k = 2–5), displaying the clustering stability using 1000 iterations of hierarchical clustering based on the DEGs acquired. [file Image_3.tif]

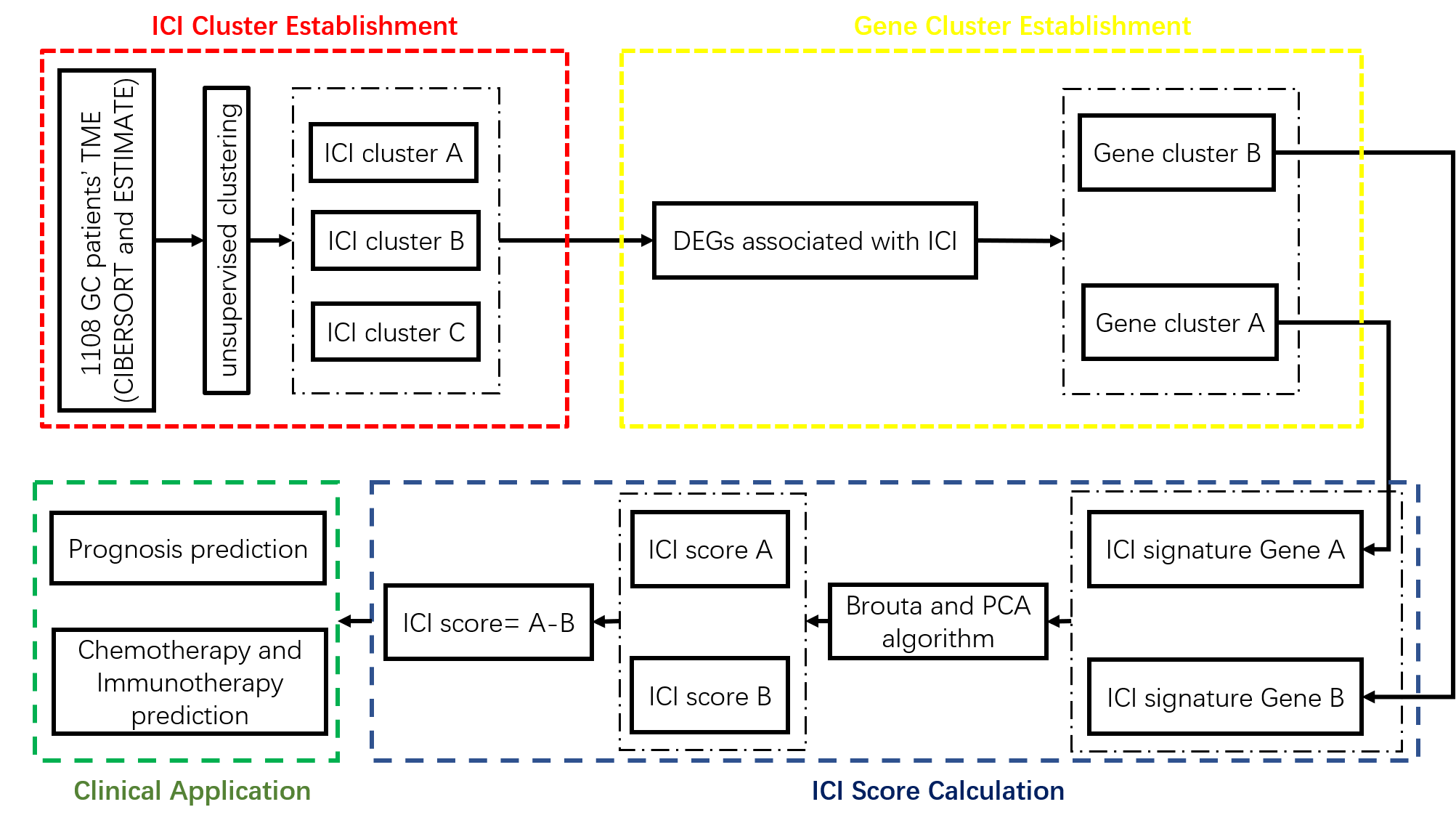

Supplement: Supplementary Figure 4 — Flowchart of this study. [file Image_4.tif]
